# SEM Observation of Hydrous Superabsorbent Polymer Pretreated with Room-Temperature Ionic Liquids

**DOI:** 10.1371/journal.pone.0091193

**Published:** 2014-03-12

**Authors:** Tetsuya Tsuda, Eiko Mochizuki, Shoko Kishida, Kazuki Iwasaki, Katsuhiko Tsunashima, Susumu Kuwabata

**Affiliations:** 1 Department of Applied Chemistry, Graduate School of Engineering, Osaka University, Suita, Osaka, Japan; 2 Department of Material Science, Wakayama National College of Technology, Gobo, Wakayama, Japan; RMIT University, Australia

## Abstract

Room-temperature ionic liquid (RTIL), which is a liquid salt at or below room temperature, shows peculiar physicochemical properties such as negligible vapor pressure and relatively-high ionic conductivity. In this investigation, we used six types of RTILs as a liquid material in the pretreatment process for scanning electron microscope (SEM) observation of hydrous superabsorbent polymer (SAP) particles. Very clear SEM images of the hydrous SAP particles were obtained if the neat RTILs were used for the pretreatment process. Of them, tri-*n*-butylmethylphosphonium dimethylphosphate ([P_4, 4, 4, 1_][DMP]) provided the best result. On the other hand, the surface morphology of the hydrous SAP particles pretreated with 1-ethyl-3-methylimidazolium tetrafluoroborate ([C_2_mim][BF_4_]) and 1-butyl-3-methylimidazolium tetrafluoroborate ([C_4_mim][BF_4_]) was damaged. The results of SEM observation and thermogravimetry analysis of the hydrous SAP pretreated with the RTILs strongly suggested that most water in the SAP particles are replaced with RTIL during the pretreatment process.

## Introduction

Room-temperature ionic liquid (RTIL) that was once called ambient-temperature molten salt and room-temperature molten salt is a liquid salt at or below room temperature [1], [2]. More specifically there are coulomb interactions between cations and anions in the liquid but not strong interactions. Due to the moderate coulomb interactions, most RTILs show negligible vapor pressure. This property is encouraging the scientists in the field of the ionic liquid to create novel vacuum technologies with ionic liquid [3]–[8]. We revealed that it is possible to observe directly RTIL itself without accumulation of electrostatic charge by a common scanning electron microscope (SEM) observation technique that is one of useful vacuum technologies [9]. In addition to this, we found that a thin RTIL layer spread on insulating materials works as a conductive layer like a thin Au layer when such materials are observed by using a common SEM system [10]–[13]. Since our first report [9], a number of similar works have been reported by various research groups [14]–[20].

Although SEM image that can give useful information on surface morphology visually has been widely employed in various fields, a common SEM system has to keep dry and high vacuum condition (∼10^−3^ Pa) in the sample chamber during the SEM observation to detect secondary electrons emitted by primary electron beam irradiation to a specimen without scattering by gaseous impurity molecules in the chamber [21], [22]. Therefore, the specimen for SEM observation must have nonvolatility as well as ability of electrostatic charge release. If a target specimen does not meet the conditions, we have to carry out a painstaking pretreatment process prior to the SEM observation. Or a cryoelectron microscope system is required, but it is not a common equipment [21], [22]. Recently we proposed a novel easy-to-use and quick approach to avoiding the awful pretreatment process [13]. By the pretreatment process consisting of glutaraldehyde fixation (if need arises) and RTIL treatment of the target sample, SEM images of the various biological specimens, e.g., pollens and insects, are obtained and their images are essentially identical to or often surpass those taken by conventional pretreatment methods [13]–[19]. However there is a limited number of researches on SEM observation of hydrous materials pretreated with RTIL [13], [18], [20]. As far as we know, hydrous polymer materials are not observed by the RTIL-used approach. In this article, we report an effective and convenient pretreatment method with RTIL for SEM observation of hydrous superabsorbent polymer (SAP) that is a very important functional polymer material for disposable hygiene products etc. The aim of this study is to establish the RTIL-used pretreatment process for SEM observation of hydrous SAP particles, to clarify the effect of RTIL species and RTIL concentration in aqueous solutions on the SEM image, and to reveal the cause of morphology preservation of the hydrous SAP particles under high vacuum condition.

## Materials and Methods

### Materials

**Figure 1:**
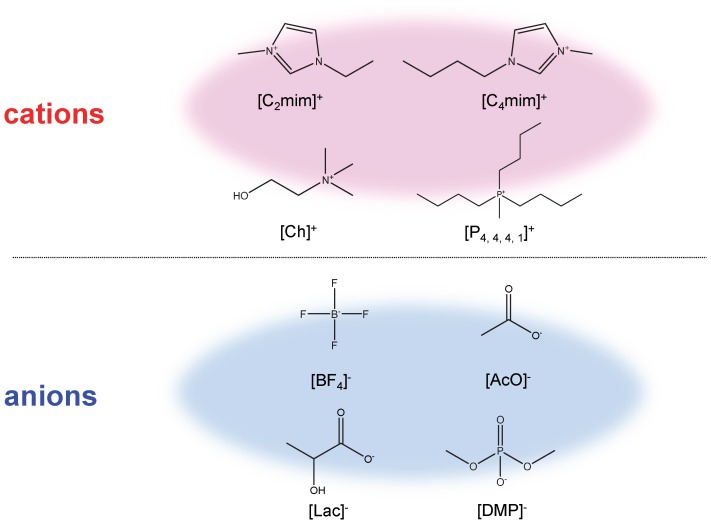
Chemical structures and abbreviation forms of the cations and anions making up the RTILs used in this article.

In this study, six RTILs were employed. Those were 1-ethyl-3-methylimidazolium tetrafluoroborate ([C_2_mim][BF_4_]), 1-ethyl-3-methylimidazolium acetate ([C_2_mim][AcO]), 1-ethyl-3-methylimidazolium lactate ([C_2_mim][Lac]), 1-butyl-3-methylimidazolium tetrafluoroborate ([C_4_mim][BF_4_]), choline lactate ([Ch][Lac]), and tri-*n*-butylmethylphosphonium dimethylphosphate ([P_4, 4, 4, 1_][DMP]). Chemical structures of the cations and anions contained in these RTILs are depicted in Figure 1. The preparation and purification processes for the RTILs are described in our previous papers [1], [2], [13], [23], [24]. Physical properties of the RTILs used are summarized in Table 1. Their density and viscosity were measured by a Kyoto Electronics Manufacturing DA-640 density meter and a Kyoto Electronics Manufacturing EMS-1000 viscometer, respectively.

**Table 1:** Physicochemical properties of the RTILs used in this article.

RTILs	Formula weight/g mol^−1^	Viscosity/cP	Density/g cm^−3^	Hydrophilicity	References
[C_2_mim][BF_4_]	197.97	36.93 (298 K)	1.2799 (298 K)	○	[25]
[C_2_mim][AcO]	170.20	143.61 (298 K)	1.099 (298 K)	○	[26]
[C_2_mim][Lac]	200.23	184.0 (303 K)	1.143 (298 K)	○	This work
[C_4_mim][BF_4_]	226.03	75 (303 K)	1.20 (303 K)	○	[27]
[Ch][Lac]	193.24	895.0 (303 K)	1.140 (298 K)	○	This work
[P_4, 4, 4, 1_][DMP]	342.39	439 (298 K)	1.03 (303 K)	○	[24]

**Figure 2:**
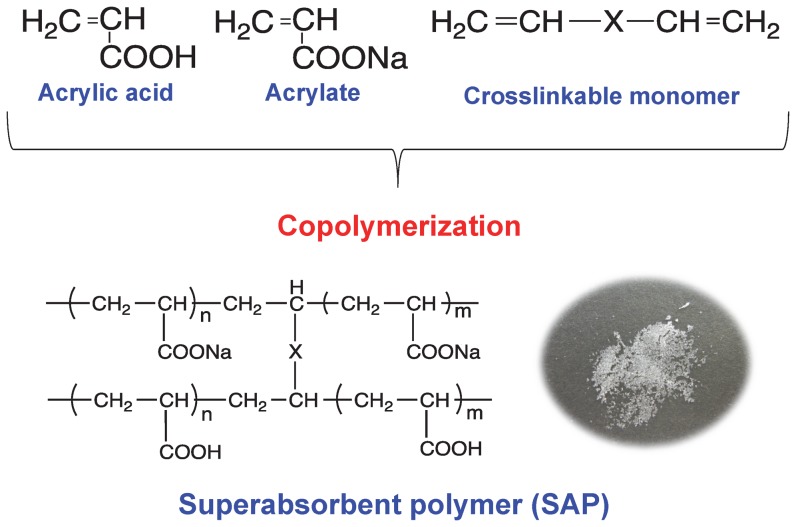
Chemical structures of the superabsorbent polymer used in this investigation.

Commercially available SAP particles prepared by copolymerization of mixtures of acrylic acid, acrylate, and crosslinkable monomer were provided by Nippon Shokubai Co., Ltd. (Japan). Common chemical structure of the SAP particles employed in this investigation is shown in Figure 2.

### Pretreatment process for SEM observation

**Figure 3:**
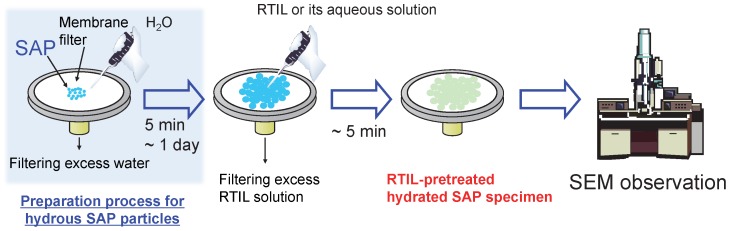
A schematic drawing of the pretreatment protocol for SEM observation of SAP specimens used in this investigation.

Pretreatment protocol of dry and hydrous SAP particles for SEM observation is illustrated in a schematic way in Figure 3. It was designed on the basis of the results reported in our previous papers [10]–[13]. The biggest advantage is that the protocol is completed with a very simple process less than 5 min and does not require any special equipment. As discussed in more detail in Results and Discussion section, most water in hydrous SAP particles is replaced with RTIL having negligible vapor pressure in the pretreatment process. It results in keeping the original shape under vacuum condition. That is, RTIL has two important tasks that are formation of electron conductive layer onto SAP samples and morphology preservation of hydrous SAP particles.

### SEM observation and thermogravimetry analysis

SEM observation was carried out by a Hitachi S-3400N scanning electron microscope (SEM) system. Secondary electron image mode was used for the observation. Prior to the SEM observation, we checked the morphology of SAP particles by a Keyence VHX-2000 digital optical microscope with a Keyence VHZ500R/W lens. Thermogravimetry analysis (TGA) of SAP particles used in this study was performed by a Bruker TG-DTA2000SA under dry nitrogen atmosphere at 10 K min^−1^. The instrument was controlled with a Bruker MTC1000SA workstation utilizing a Bruker WS5003 software. Aluminum pan for the analysis was employed, and the specimen was prepared in an argon gas-filled grove box (Vacuum Atmosphere Company, NEXUS II system).

## Results and Discussion

### Morphological observation by a digital optical microscope

**Figure 4:**
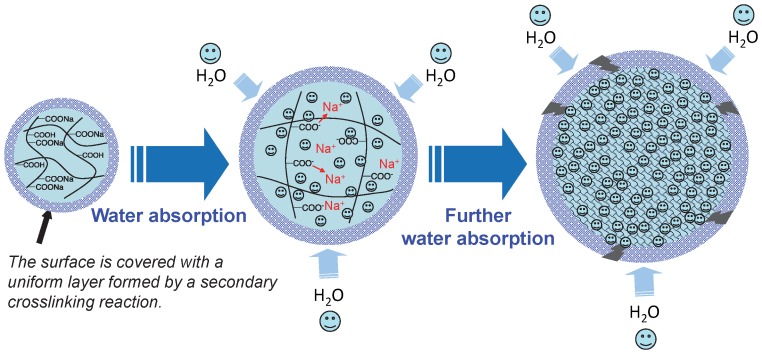
Schematic illustration of water absorption process of a SAP particle.

**Figure 5:**
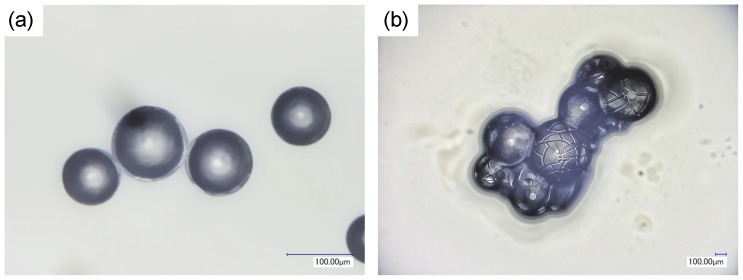
Digital optical microscope images of (a) original dry and (b) hydrous SAP particles. The images were taken under atmospheric condition.

Schematic illustration of water absorption process of a SAP particle is depicted in Figure 4. The SAP particle used was covered with a uniform layer formed by a secondary crosslinking reaction to keep the spherical structure after water absorption. However if the SAP particle absorbs too much water, it is well-known that many cracks appear on a top layer of the particle. Figure 5 indicates digital microscope images of the SAP particles before and after water absorption. Particle size of the dry and water-absorbed SAP ones were about 85∼110 μm and 300∼550 μm, respectively. In the latter case, many cracks caused by their significant volume expansion were recognized on the surface. But we could not observe fine structures of the particles because of the limitation of optical microscope.

### Morphological observation by a SEM microscope

**Figure 6:**
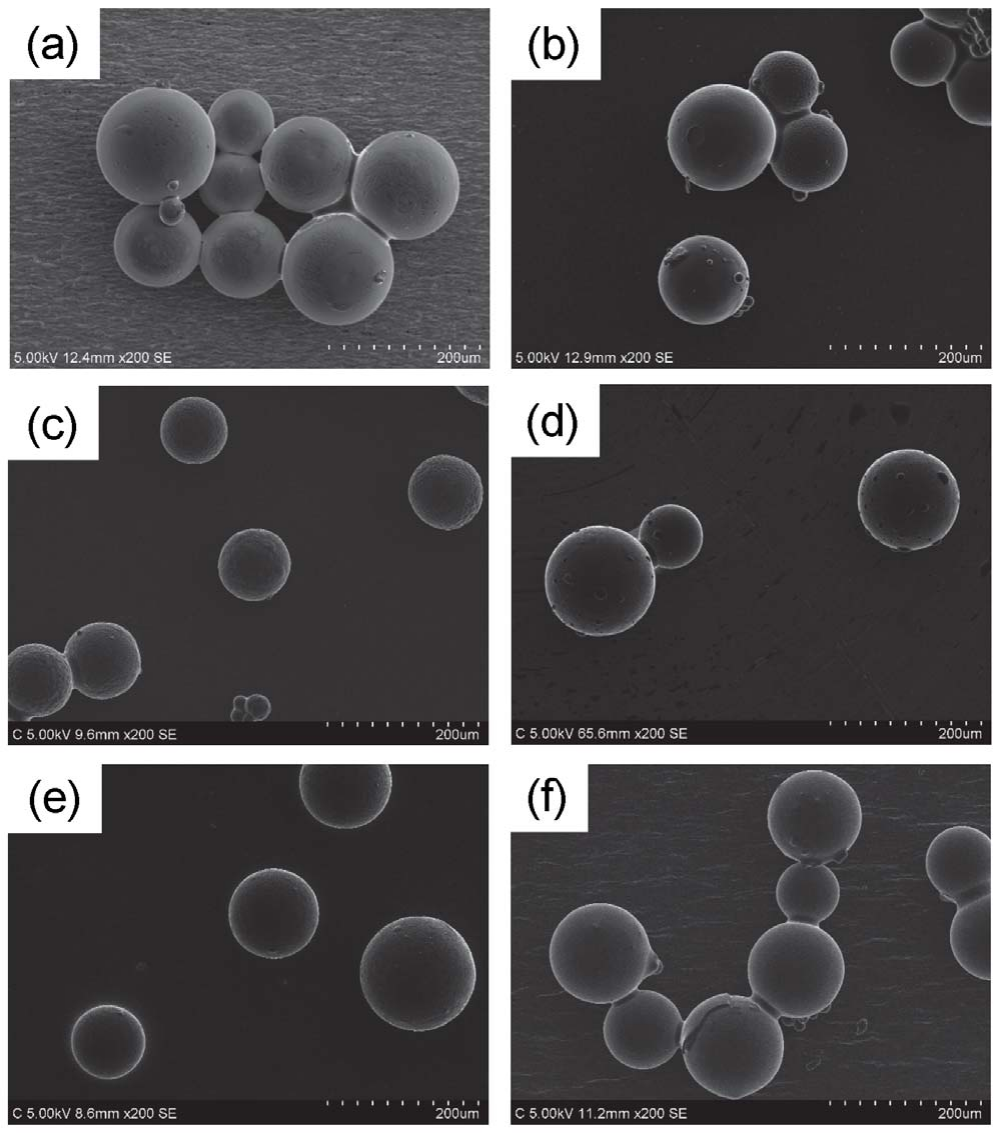
SEM images of original dry SAP particles pretreated with different neat RTILs. (a) [C_2_mim][BF_4_]; (b) [C_2_mim][AcO]; (c) [C_2_mim][Lac]; (d) [C_4_mim][BF_4_]; (e) [Ch][Lac]; (f) [P_4, 4, 4, 1_][DMP].

**Figure 7:**
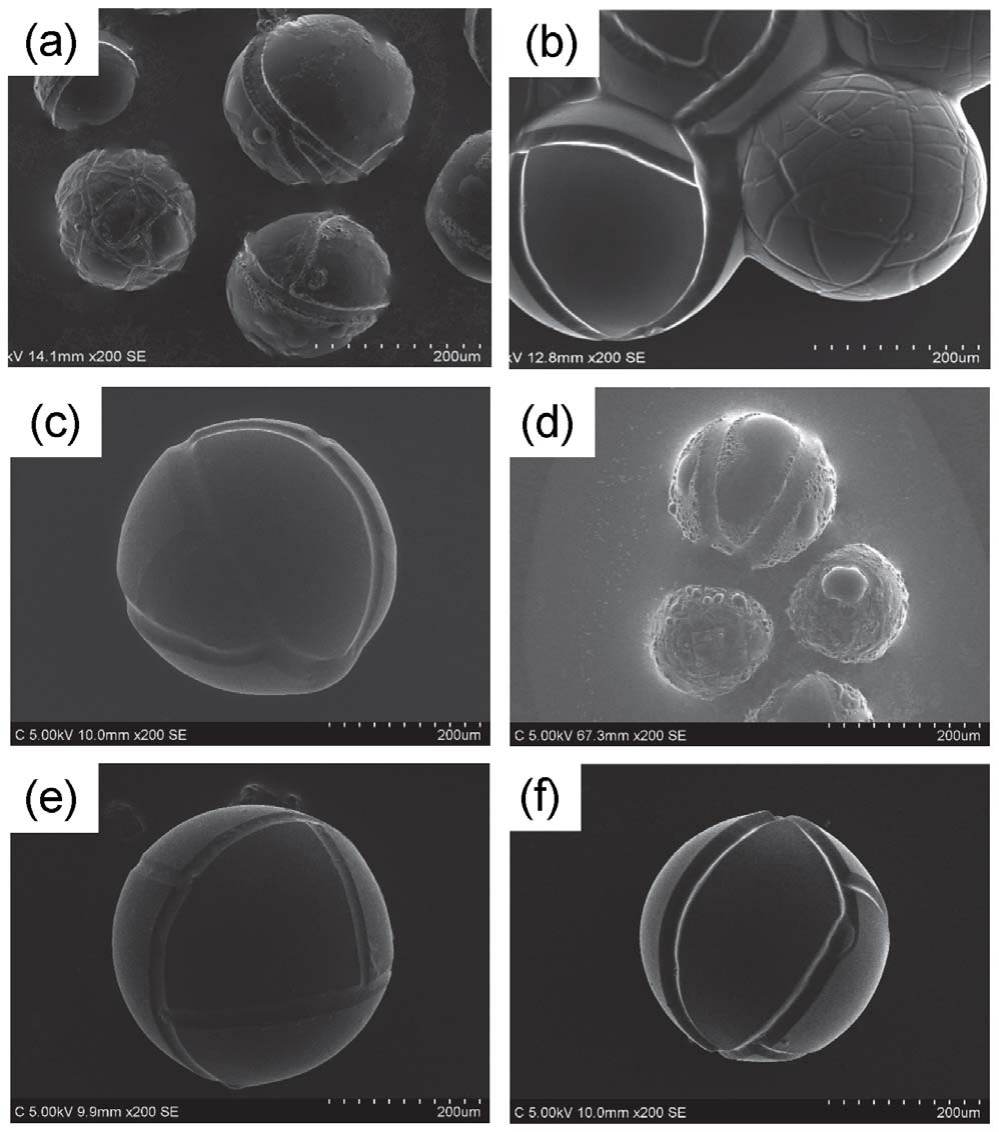
SEM images of hydrous SAP particles pretreated with different neat RTILs. (a) [C_2_mim][BF_4_]; (b) [C_2_mim][AcO]; (c) [C_2_mim][Lac]; (d) [C_4_mim][BF_4_]; (e) [Ch][Lac]; (f) [P_4, 4, 4, 1_][DMP].

Figure 6 depicts SEM images of original dry SAP particles pretreated with six types of neat RTILs. The pretreatment was conducted by the scheme in Figure 3 but the water absorption step was skipped. Of course before the pretreatment we could not obtain SEM images without accumulation of electrostatic charge at the surface, but it is not the case with the RTIL-coated SAP particles. We could observe all the specimens without any difficulty. There was little change in quality of the SEM images. Particle size of the dry SAP particles determined from the SEM images was essentially identical to that from the digital optical microscope (Figure 5a). Even if the dry particles were immersed in the RTILs for several hours, the size did not alter. In other words, the RTILs were not absorbed into the SAP particles. However, as shown in Figure 7 if the water-absorbed SAP particles were pretreated with the process using neat RTILs in Figure 3, the morphology changed depending upon the RTIL species. In the case of [C_2_mim][AcO], [C_2_mim][Lac], [Ch][Lac], and [P_4, 4, 4, 1_][DMP], we could observe fine structures including cracks like the digital optical microscope image shown in Figure 5b and a high-magnification SEM image without difficulties, too (Figure S1). Especially the pretreatment with the neat [C_2_mim][AcO] and [P_4, 4, 4, 1_][DMP] provided clear SEM images with a high contrast ratio. As given in Table 1, these two RTILs have relatively-low density. In general, penetration depth of the primary electron beam is inversely proportional to the density of the target material [22], i.e., when the [C_2_mim][AcO] and [P_4, 4, 4, 1_][DMP] are exploited for the scheme in Figure 3, secondary electrons are easily emitted from SAP particles by the primary electron bombardment compared to the [C_2_mim][Lac] and [Ch][Lac]. It is the reason why the clear SEM images were obtained after the pretreatment with the [C_2_mim][AcO] and [P_4, 4, 4, 1_][DMP]. Each particle size of the hydrous SAP after the pretreatment, except [C_2_mim][BF_4_] and [C_4_mim][BF_4_], was about 330∼370 μm. It is slightly smaller than that estimated from the digital optical microscope image, suggesting that a limited amount of water evaporated from the SAP particles. However, once the evaporation behavior reached a stable state, the surface morphology and the particle size remained independently of the time placed in a SEM chamber (Figure S2). Meanwhile, as to the specimens pretreated with [C_2_mim][BF_4_] and [C_4_mim][BF_4_], the surface morphology varied awkwardly and the particle size became obviously smaller than that for other four RTILs. Hydrolysis reaction of [BF_4_]^-^ anion is known to occur under presence of water [28], and it is enhanced at higher temperature. We think hydrogen fluoride and/or byproducts yielded during the hydrolysis reaction caused the unexpected results. Similar behavior was also confirmed when the cultured mouse-derived fibroblast L929 cells pretreated with RTILs was observed by a common SEM [13]. Figure S3 indicates SEM images of hydrous SAP particles at different water absorption time after the pretreatment process using [C_2_mim][AcO]. As we expected, the particle size became larger with the water absorption time. Cracks on the surface did not appear if the particle size was less than ca. 200 μm.

**Figure 8:**
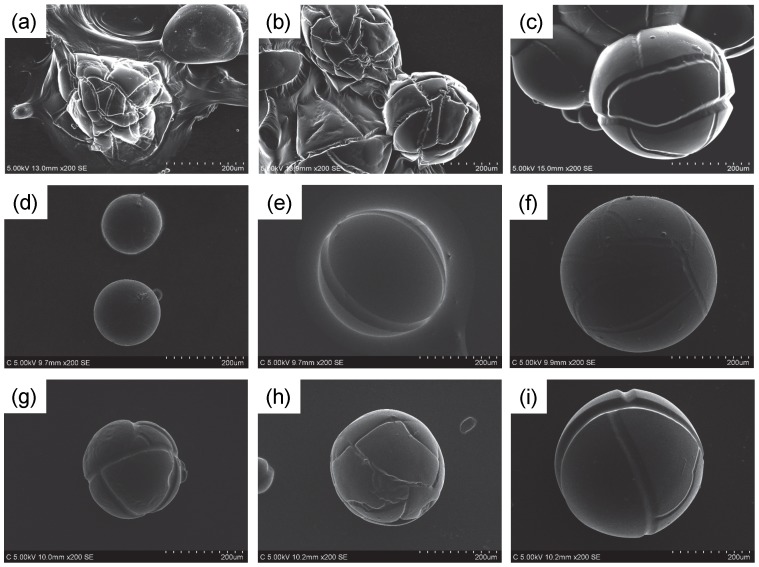
SEM images of hydrous SAP particles pretreated with different RTIL aqueous solutions. (a–c) [C_2_mim][OAc]; (d–f) [Ch][Lac]; (g–i) [P_4, 4, 4, 1_][DMP]. The RTIL concentrations in the aqueous solutions were (a, d, g) 10 vol %, (b, e, h) 50 vol %, (c, f, i) 100 vol %. The immersion time of the SAP in water was overnight.

We found some neat RTILs work very well for SEM observation of the hydrous SAP particles. Because RTIL is often diluted with water and ethanol for the RTIL-based pretreatment process to form a uniform thin layer on the target specimens [10]–[13], we also investigated the effect of the RTIL concentration in aqueous RTIL solution on SEM images of water-absorbed SAP particles. Figure 8 shows SEM images of the water-absorbed SAP pretreated with different RTIL aqueous solutions. Here we used three types of RTILs, [C_2_mim][OAc], [Ch][Lac], and [P_4, 4, 4, 1_][DMP]. For the [C_2_mim][OAc], the hydrated SAP particles were severely damaged by using the aqueous solutions with 10∼50 vol.% [C_2_mim][OAc]. As regards the [Ch][Lac] and [P_4, 4, 4, 1_][DMP], severe damages were not observed but the particle size decreased with decreasing the RTIL concentration owing to the insufficient substitution of water to RTIL in the SAP particles. These results suggest that the key to obtain desirable SEM image by the pretreatment process shown in Figure 3 is to use neat RTILs except [BF_4_]^−^-based ones.

### Thermogravimetry analysis of hydrous SAP particles

**Figure 9:**
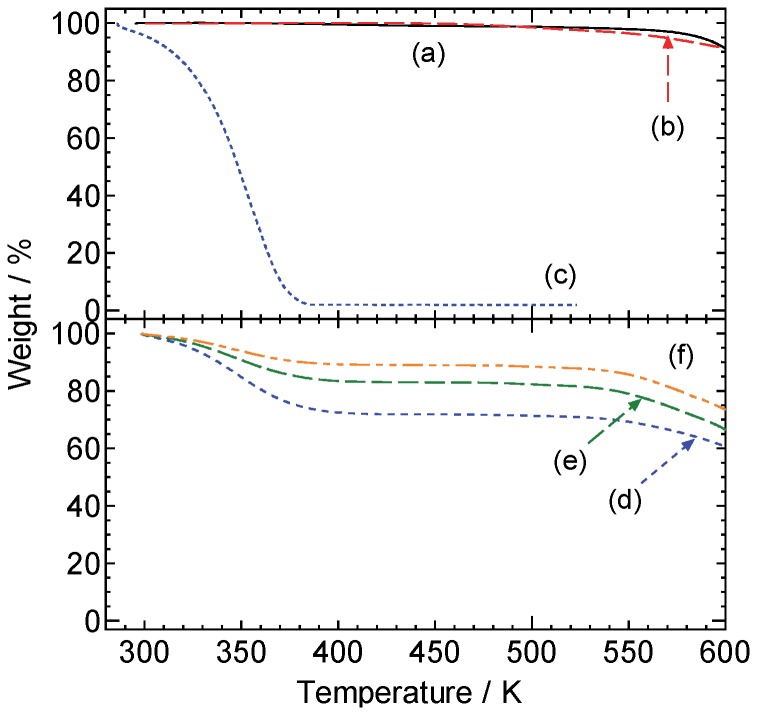
Results of thermogravimetry analyses. (a) [P_4, 4, 4, 1_][DMP], (b) dry SAP particles, (c) hydrous SAP particles, (d) hydrous SAP particles pretreated with neat [P_4, 4, 4, 1_][DMP], (e) hydrous SAP particles pretreated with neat [P_4, 4, 4, 1_][DMP] left under vacuum condition for 3 hrs, and (f) hydrous SAP particles pretreated with neat [P_4, 4, 4, 1_][DMP] left under vacuum condition for 24 hrs. The measurements were conducted at 10 K min^−1^.

As described in a previous section, the scheme in Figure 3 is a favorable pretreatment method for SEM observation of hydrous SAP particles although we have to select RTIL carefully. The advantage is that we can know their detailed morphologies easily and rapidly. But still it is unclear whether water is contained in the particles pretreated with the scheme or not. In order to reveal it, we carried out thermogravimetry analyses as shown in Figure 9. In this analyses, we chose [P_4, 4, 4, 1_][DMP] as a test RTIL because it gave the best result in this investigation. The [P_4, 4, 4, 1_][DMP] and dry SAP showed a very high thermal stability over ca. 600 K. Weight loss of the hydrous SAP specimen without pretreatment by the scheme began soon after the initiation of the analysis and it continued until the temperature reached ca. 380 K. It should be due to the water evaporation from the hydrous SAP particles. After pretreatment of the hydrous SAP with the neat [P_4, 4, 4, 1_][DMP], the weight loss was suppressed drastically. It diminished in accordance with the time that the specimen was left under vacuum condition, that is, water gradually evaporated from the specimen. It is reasonable to assume that the weight loss should be derived from free water contained in the RTIL layer onto the specimens, given that the particle size of the RTIL-pretreated SAP specimen before and after heating at 403 K was invariant (Figure S4). Thus, it is highly probable that the most water molecules in hydrous SAP particles are replaced with RTIL during the pretreatment process.

### Conclusions

We have succeeded in SEM observation of hydrous SAP particles by employing the easy-to-use RTIL-based pretreatment process that can be completed within 5 min. When appropriate neat RTILs were exploited for the process, very clear and spontaneous SEM images could be observed. But, in the case where the SAP specimens pretreated with 1-ethyl-3-methylimidazolium tetrafluoroborate, the specimens seemed to be slightly damaged by an undesirable hydrolysis reaction of the [BF_4_]^−^. As to RTIL aqueous solutions, it was difficult to observe the SEM images of the hydrated SAP samples, especially at lower RTIL concentration. Our results on the SEM observation and thermogravimetry analysis of the hydrous SAP particles strongly indicate that most water in the SAP particles are replaced with RTIL during the scheme in Figure 3. We believe that the findings on the SEM observation of polymer aqueous gel materials will be very beneficial to scientists and engineers in the field of polymer science.

## Supporting Information

Figure S1 **SEM images of hydrous SAP particles pretreated with the scheme in** **Figure 3** **.** The immersion time of the SAP in water was overnight. The RTIL used for the scheme was neat [P_4, 4, 4, 1_][DMP].
(EPS)

Figure S2 **Variation in surface morphology of a hydrous SAP particle pretreated with neat [P_4, 4, 4, 1_][DMP], when the specimen was left in a SEM chamber.** The time of standing was (a) 0 hr, (b) 2 hrs, (c) 4 hrs and (d) 7 hrs. The immersion time of the SAP in water was overnight.
(EPS)

Figure S3 **SEM images of hydrous SAP particles prepared at different water absorption time.** The specimens were pretreated with neat [C_2_mim][OAc]. The immersion time of the SAP in water was (a) 0 min, (b) 5 min and (c) overnight.
(EPS)

Figure S4 **SEM images of hydrous SAP particles pretreated with scheme in** **Figure 3** **(a) before and (b) after heat treatment at 403 K for 20 min.** The RTIL used for the scheme was neat [P_4, 4, 4, 1_][DMP].
(EPS)
